# Supplementary material for: CryoEM reveals the structure of an archaeal pilus involved in twitching motility
Source: Nat Commun. 2024 Jun 14;15:5050. doi: 10.1038/s41467-024-45831-w (PMC11178815; doi:10.1038/s41467-024-45831-w)
Supplement: Supplementary file 1 — Supplementary Figs. [file 41467_2024_45831_MOESM1_ESM.pdf]

Supplementary Figure 1

a

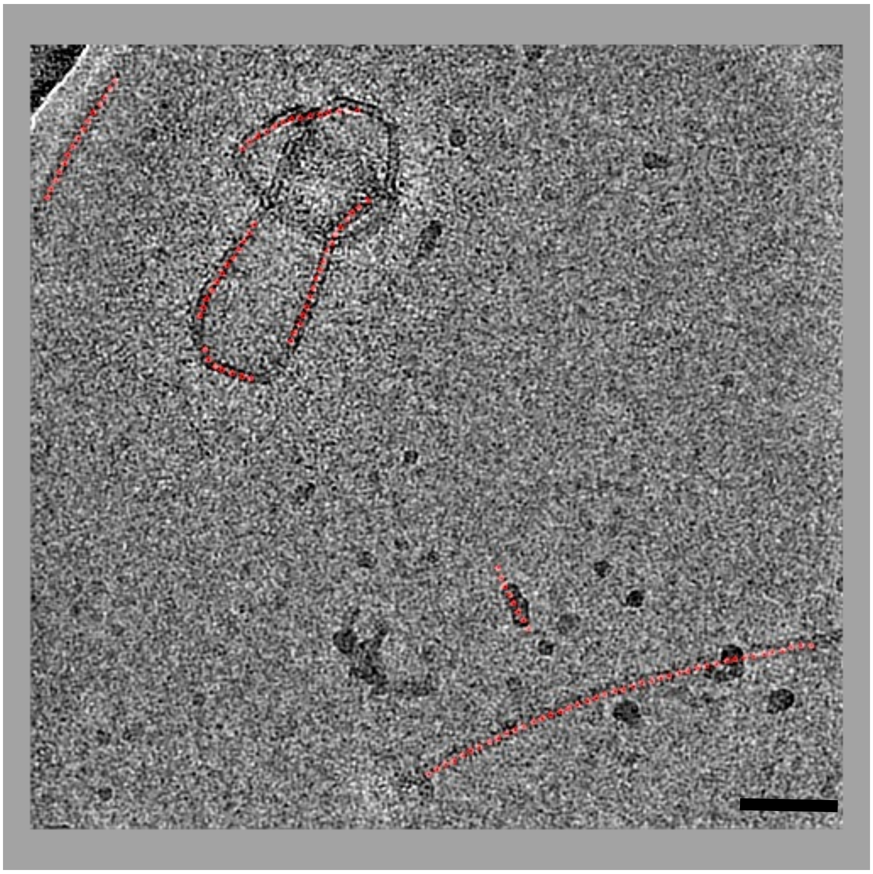

b

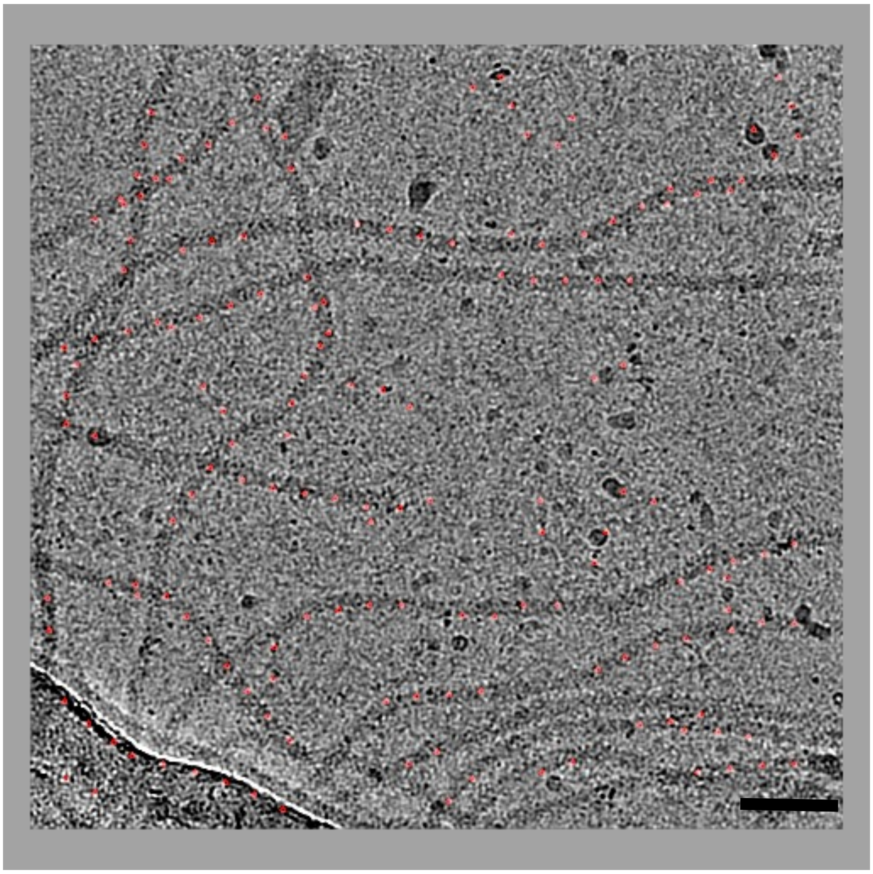

Supplementary Figure 1 – CryoEM of isolated Aap

a, b, raw micrographs of isolated and vitrified Aap indicate that Aap can adopt high degrees of curvature. Scale bar 500nm

Supplementary Figure 2

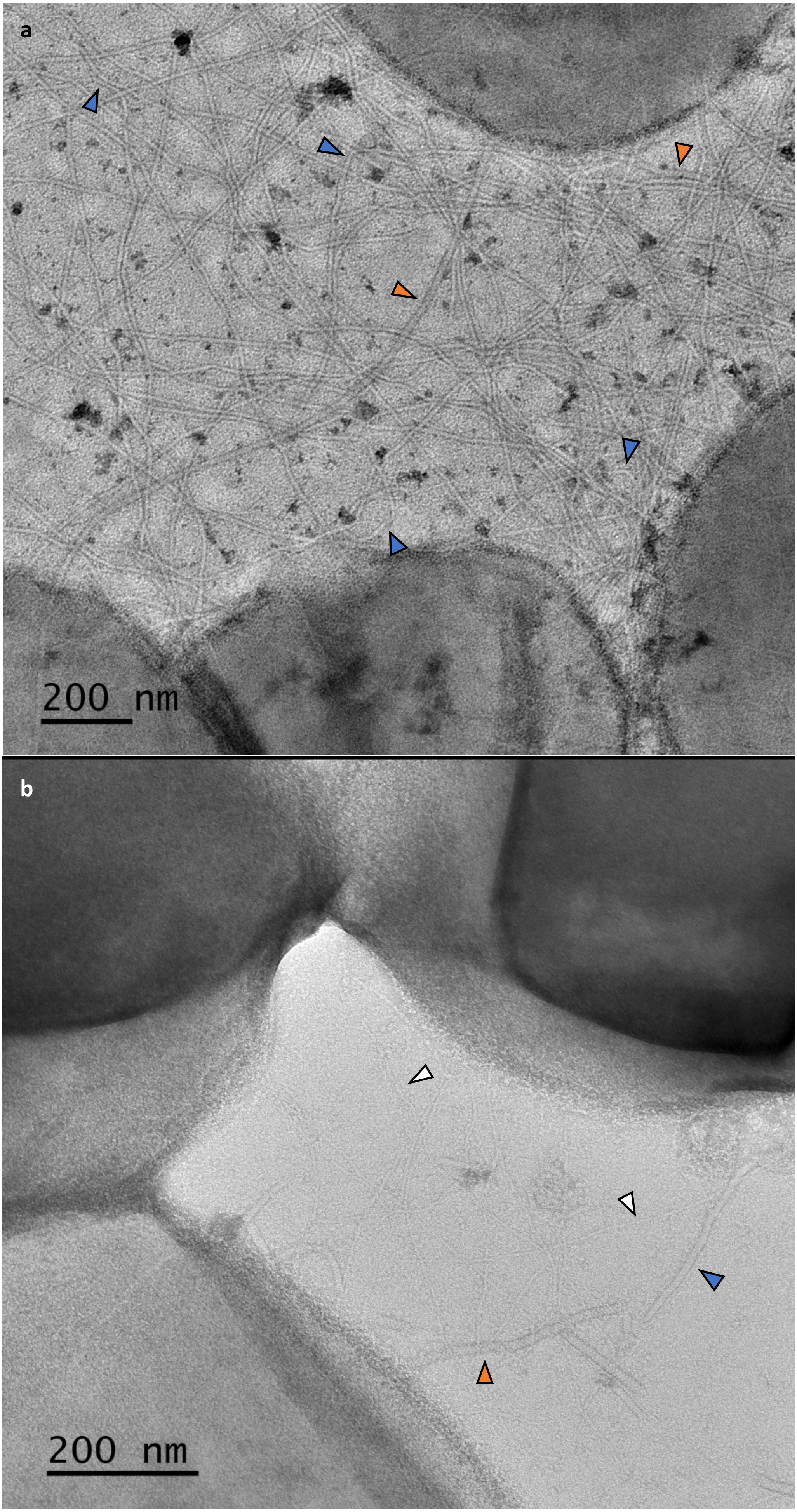

Supplementary Figure 2 – Negative stain EM of *wt S. acidocaldarius*

a, b, electron micrographs of negatively stained *S. acidocaldarius wt* (MW001) cells. Aap (blue arrowheads; a and b), archaella (orange arrowheads; a and b) and threads (white arrowheads; b) can be distinguished by diameter and apparent stiffness. Aap have a diameter of ~8 nm[2] and are highly curved. Archaella are ~12 nm[1] wide and undulate slightly. Threads measure ~4 nm[3] in diameter and appear relatively straight.

Supplementary Figure 3

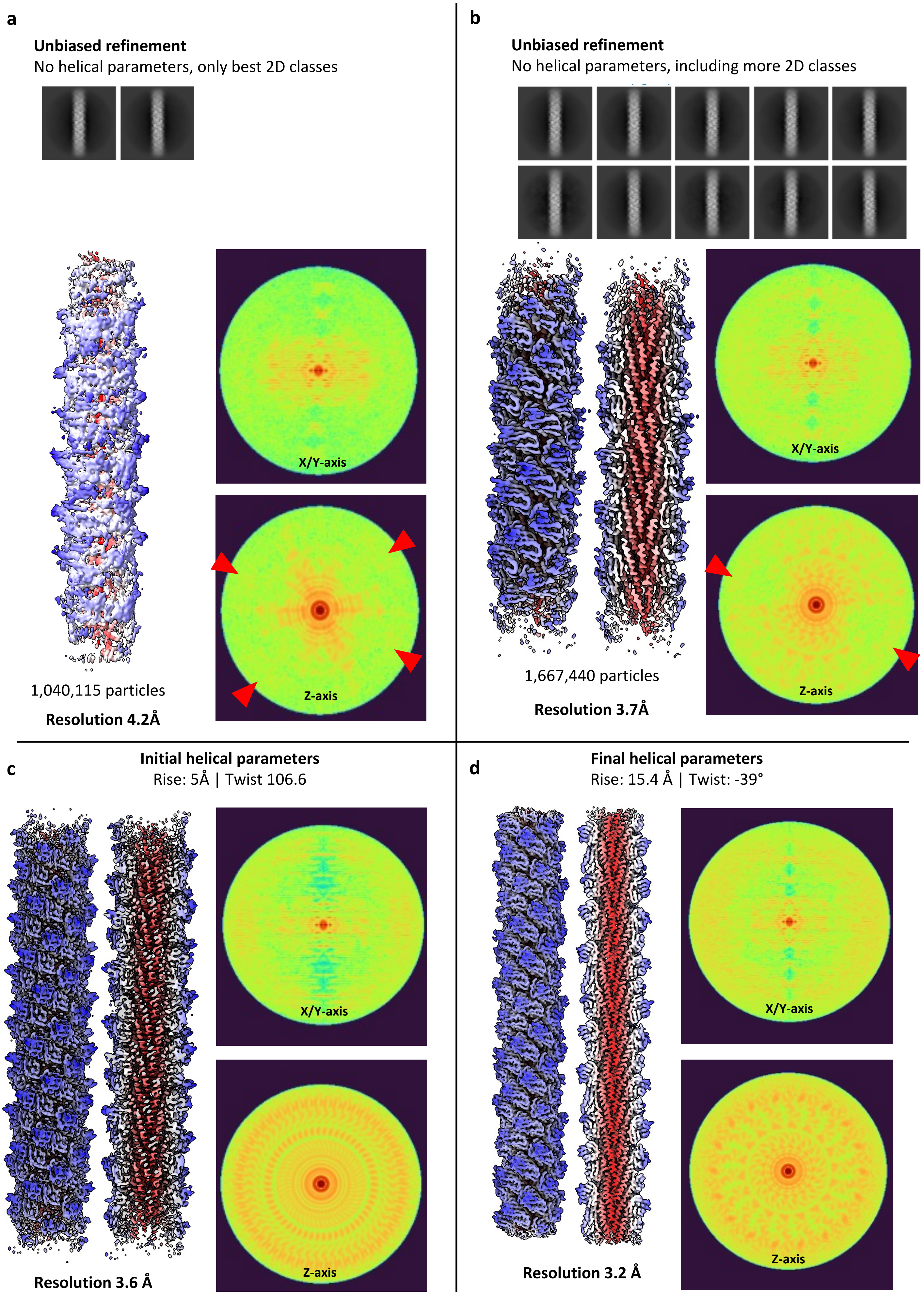

Supplementary Figure 3 – Unbiased helical refinement of the *S. acidocaldarius* Aap

a, using the two best 2D classes for the initial unbiased 3D refinement (without applying helical parameters) resulted in a 4.2 Å map with clear artifacts, resulting from a limited number of views. This was evident by a lack in information in Fourier space. The Power spectrum related to the side of the Aap lacked information at high spatial frequencies (top), and the Power spectrum related to the end-on view of the filament showed wedges of missing information (bottom, red arrowheads). **b**, adding more 2D classes (including less well resolved ones), resulted in an improved unbiased 3D refinement with 3.7 Å resolution. While there was still some missing information in the power spectra (red arrowheads), molecular details could be discerned, initially suggesting a helical rise of 5 Å and twist of 106.6°. **c**, Applying these parameters in a new round of helical refinement resulted in a map with nominally increased resolution (3.6 Å). However, the core α-helices were not well resolved and could thus not be modelled. Further inspection and initial model building based on the unbiased map (b) showed that the Aap was composed of subunits adopting three different conformations, revealing more accurate helical parameters of 15 Å rise and -39° twist. Applying these parameters to helical refinement improved the resolution further to 3.2 Å (**d**). Note that the power spectra in (c), which correspond to the initial helical parameters (5 Å rise and 106.6° twist) showed clear oversampling compared to the unbiased ones (b). In contrast, the power spectra of the corresponding to map were 15 Å rise and -39° twist were applied (**d**) enhanced the power spectra of the unbiased map (**b**).

Supplementary Figure 4

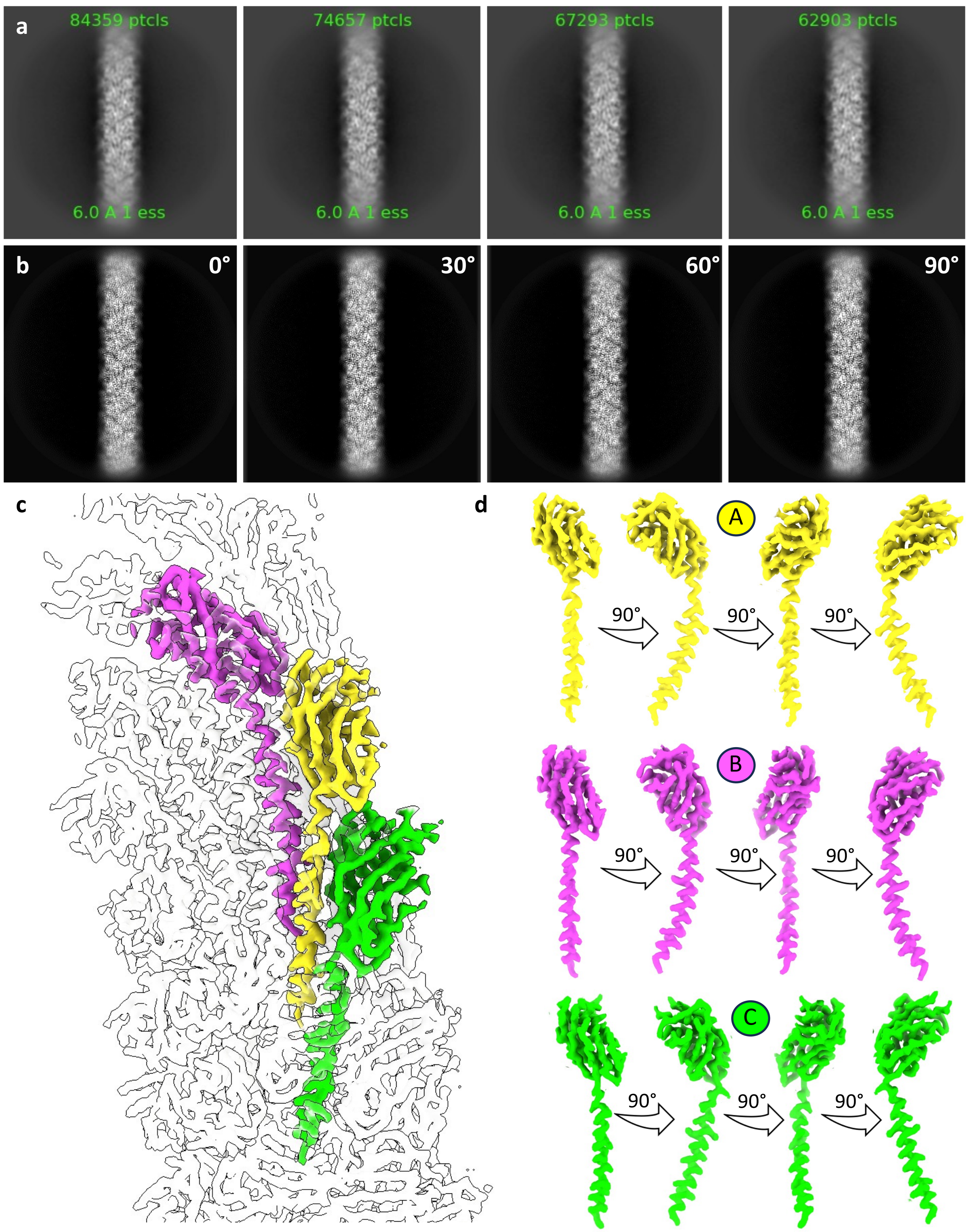

Supplementary Figure 4 – Updating helical parameters improves the resolution of the map

a, example 2D classes of the Aap from CryoSPARC; **b**, projections of the final map of the Aap rotated around the filament's long axis by 0°, 30°, 60° and 90°. **c**, 3.2 Å resolution map of the *S. acidocaldarius* Aap, with three AapB subunits highlighted (yellow, conformation A; magenta, conformation B; green, conformation C). **d**, various views of the three AapB conformations showing the quality of the map.

Supplementary Figure 5

a

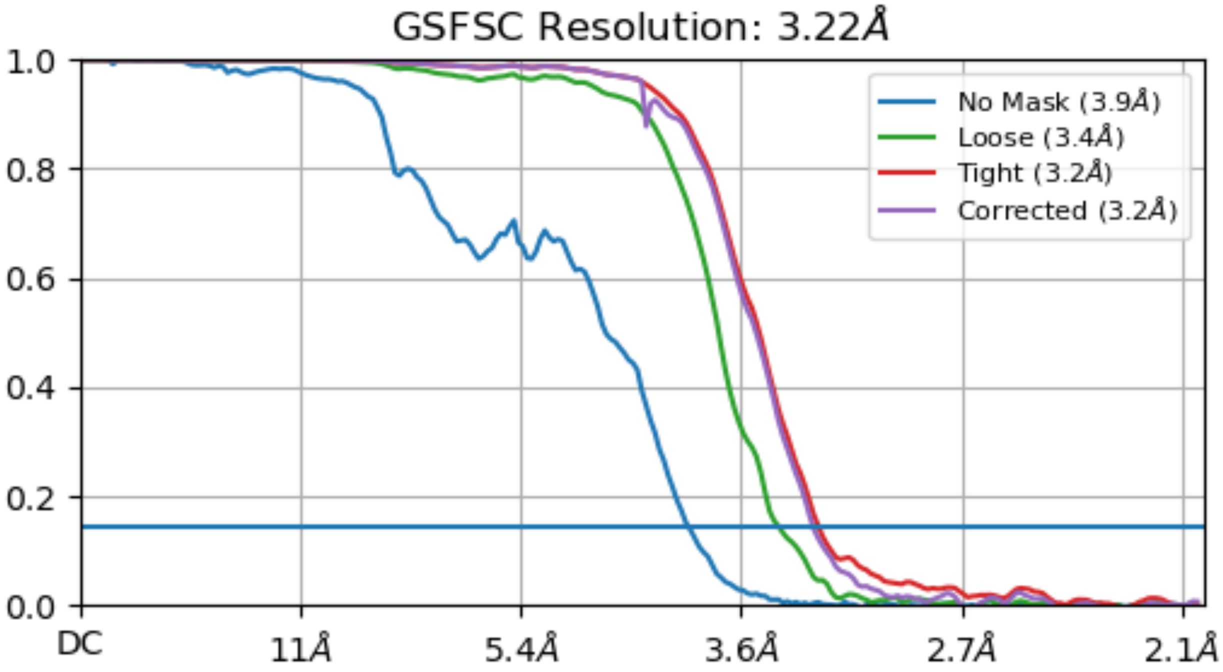

b

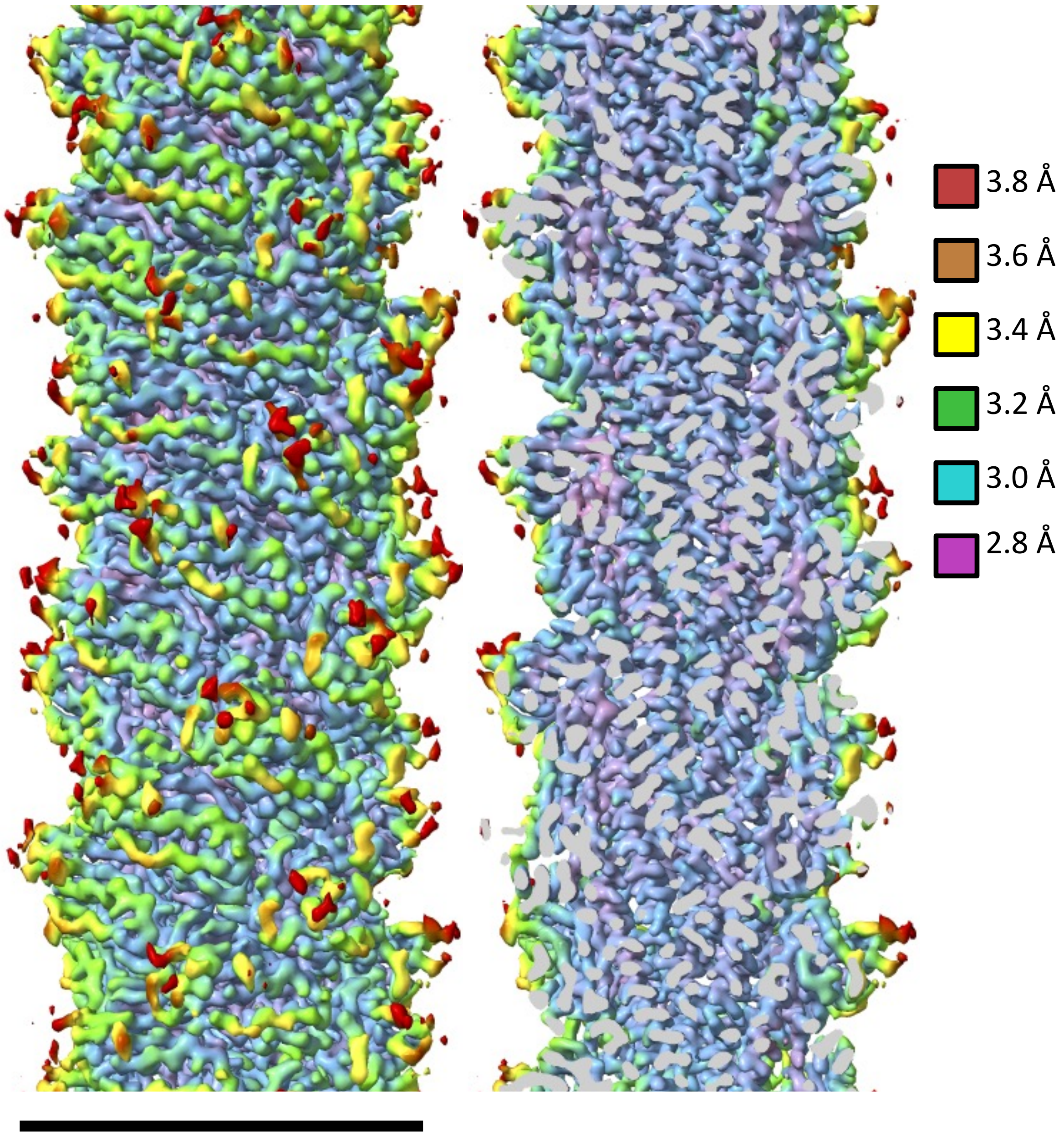

Supplementary Figure 5 – Resolution estimation

a, Fourier Shell Correlation (FSC) of the *S. acidocaldarius* Aap map indicating a global resolution of 3.2 Å. **b**, local resolution estimation suggests that the core of the filament reaches a resolution value of ~2.8 Å, while the periphery of the filament (including the surface glycans) is less well resolved (~3.8 Å). Scale bar 80 Å.

Supplementary Figure 6

a

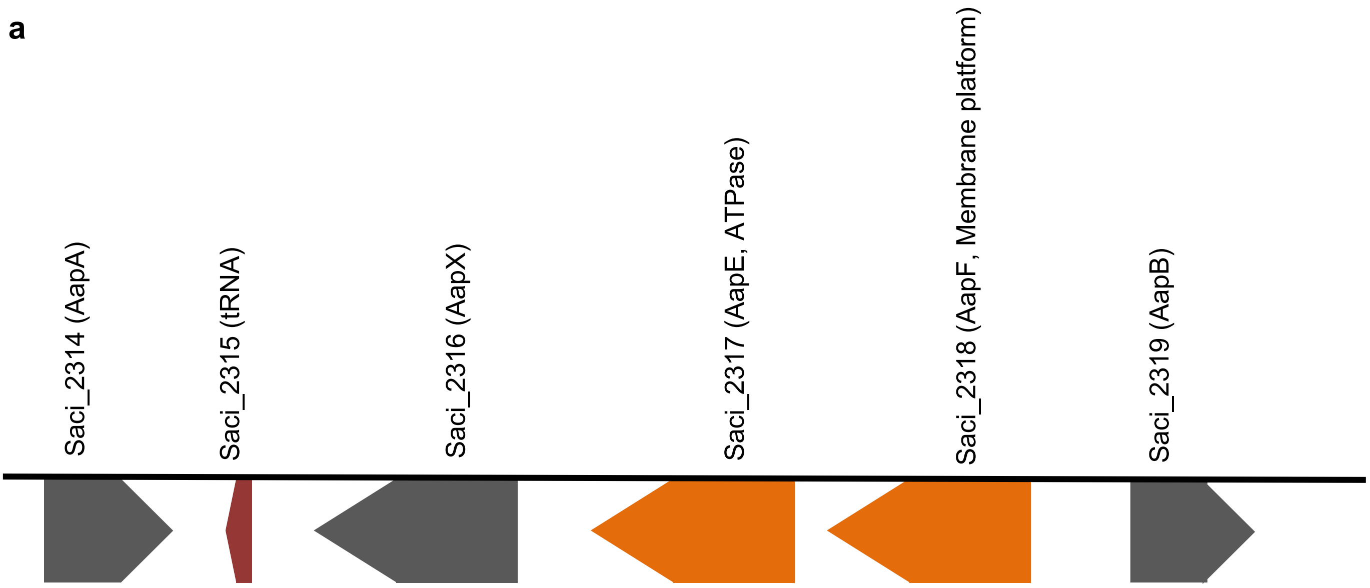

b

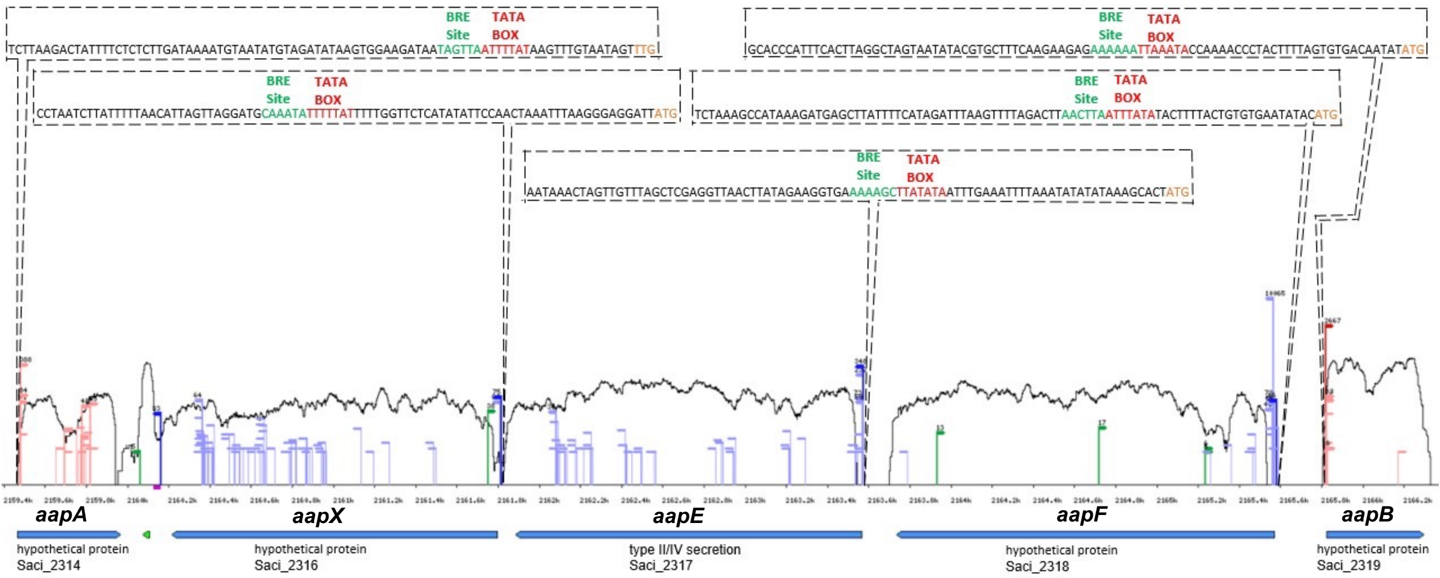

Supplementary Figure 6 – Aap gene cluster and transcriptomics profile
a, Schematic of the Aap gene cluster in *S. acidocaldarius*, encoding for the genes *aapA*, *aapX*, *aapE*, *aapF* and *aapB*. **b**, RNA-sequencing profiles for each gene of the *aap* cluster. BRE sites within promotor regions are highlighted in green, TATA boxes in red [1].

Supplementary Figure 7

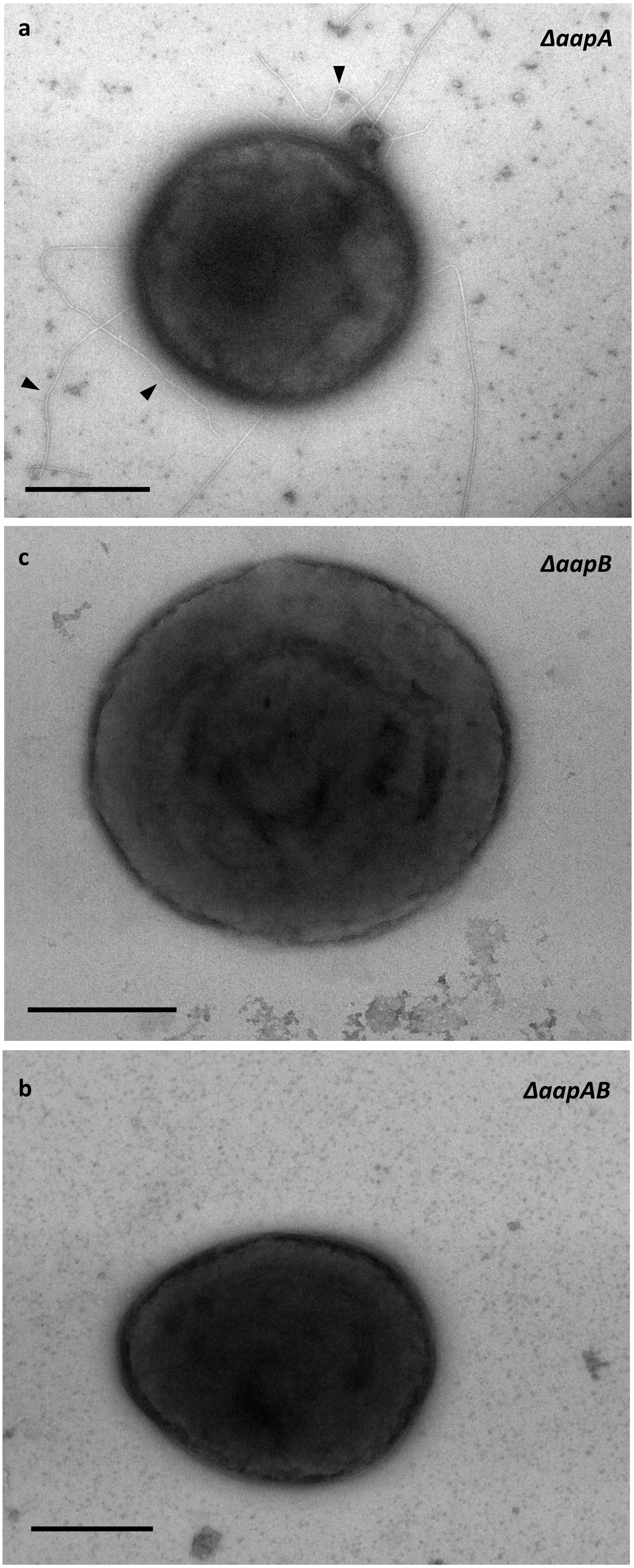

Supplementary Figure 7 – Negative stain electron microscopy of *S. acidocaldarius* mutants

a, the Δ*aapA* knockout strain (MW153) still assembles AAP (arrowheads), while Δ*aapB* (MW154) **(b)** and Δ*aapAB* double knockout (MW161) (**c**) do not produce Aap. Black arrowheads represent Aap filaments

Supplementary Figure 8

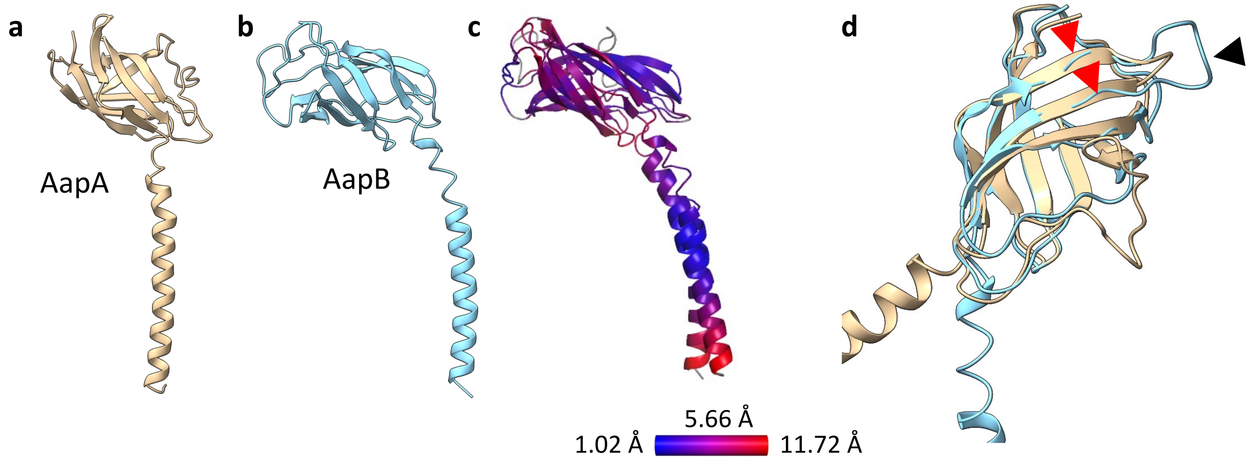

e

```
AapB            -----MNIEVKKSKKKNMRALSGAIVALILVIAGVIIAIAVVLFAFGLIPGISNQGSIQV  55
AapA            MYNKITMISRYRYDKRRIRALSGAIVALILVIAGVIIATAVVLFAFGLIPAISNQGSAQV  60
                       *.  : .*:.:******************** ***********.****** **

AapB            LGSGTITNSTASGSSRTIYNITITVKNTGT-TSISVTSININGQPFNING----------  104
AapA            VGTGAIEQAGS-----GQYNIIITVRNTASNFNVSVTSINIAGISFTINKINNITYNPNN  115
                :*:*:* :: :       *** ***:**.:  .:******* *  *.**

AapB            TAPSIPAGRTQPITFEVTPASGKPNFSPGASYTATIYFSNGQGAPATLIYQG  156
AapA            PMEQVGPGKTETLTITATPT-SSIVFSSGQTYTATVYFSNGLGAPTTLIYQG  166
                   .:  *:*: :*: .**: ..  ** * :****:***** ***:******
```

Supplementary Figure 8 – AapA and AapB from *S. acidocaldarius* in comparison

a, AlphaFold2 prediction of AapA (beige). **b,** experimentally determined structure AapB (blue) in A conformation. **c,** RMSD between (a) and (b) showing their structural similarity. **d,** AapA and AapB superimposed. The red arrowheads highlight β-sheets 1 and 2, which are longer in AapA than in AapB. The black arrowhead highlights a loop region that is predicted to be longer in AapB, compared to AapA. **e,** sequence alignment between AapA and AapB. Black arrows show the N-glycosylation sites in AapB and a red arrow indicates a consensus (NXS/T) N-glycosylation sequon that does not appear to be glycosylated in AapB. The blue arrows highlight predicted NXS/T N-glycosylation sequons in AapA.

Supplementary Figure 9

a **b** **c**

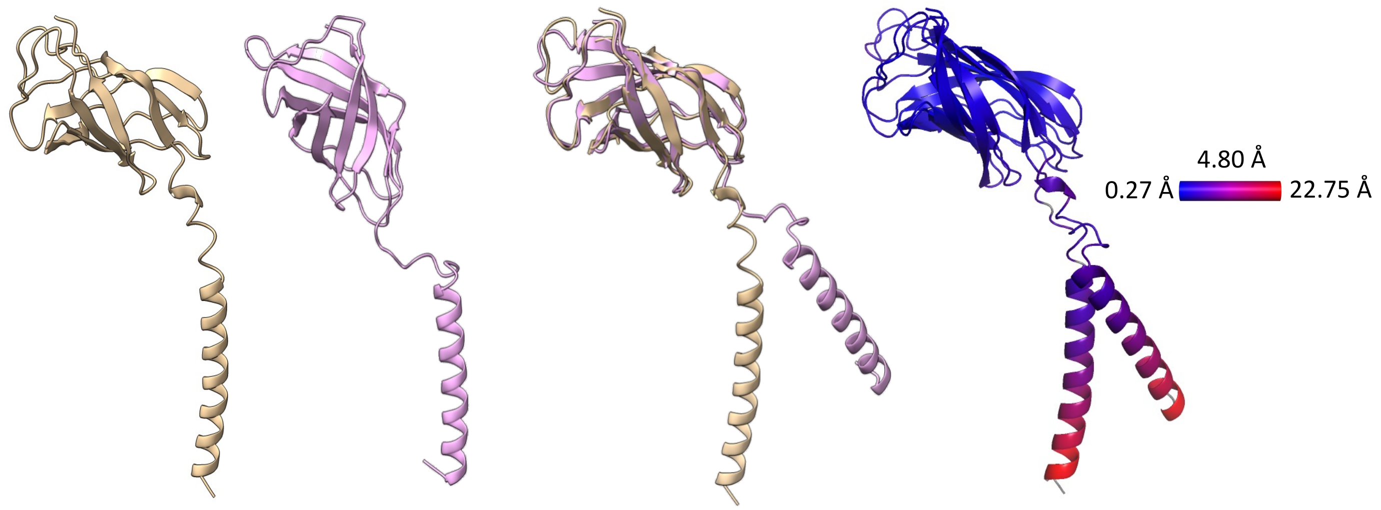

AapB (cryoEM) AapB (AlphaFold2) AapB (cryoEM vs. Alphafold2) RMSD (cryoEM vs. Alphafold2)

---

d

```
ModelAngelo  1    LDGAITALILTIAGVIIAIAVVLFAFGLIPGLSNNGCVQVLGSGEVEN-VVGGDPKVVYN  59
                  L GAI ALIL IAGVIIAIAVVLFAFGLIPG+SN G +QVLGSG + N    G  + +YN
AapB         16   LSGAIVALILVIAGVIIAIAVVLFAFGLIPGISNQGSIQVLGSGTITNSTASGSSRTIYN  75

ModelAngelo  60   ITIEVKNTGTTTVSVTSININGQPFIIRGEAPSVPAGKTQPIEFEVEPAGGKPIFSPGAS  119
                  ITI VKNTGTT++SVTSININGQPF I G APS+PAG+TQPI FEV PA GKP FSPGAS
AapB         76   ITITVKNTGTTSISVTSININGQPFNINGTAPSIPAGRTQPITFEVTPASGKPNFSPGAS  135

ModelAngelo  120  YTATIYFTNGQGAPATLIYQ  139
                  YTATIYF+NGQGAPATLIYQ
AapB         136  YTATIYFSNGQGAPATLIYQ  155
```

Identity: 108/140 (77%)
Positives: 118/140 (84%)

---

e

```
ModelAngelo  1    LDGAITALILTIAGVIIAIAVVLFAFGLIPGLSNNGCVQVLGSGEVENVVGGDPKVVYNI  60
                  L GAI ALIL IAGVIIA AVVLFAFGLIP +SN G  QV+G+G +E    G     YNI
AapA         21   LSGAIVALILVIAGVIIATAVVLFAFGLIPAISNQGSAQVVGTGAIEQAGSGQ----YNI  76

ModelAngelo  61   TIEVKNTGTT-TVSVTSININGQPFIIRG-----EAPSVPAGKTQPIEFEVEPAGGKP  112
                   I V+NT +   VSVTSINI G  F I         P+ P   +  P + E       P
AapA         77   IITVRNTASNFNVSVTSINIAGISFTINKINNITYNPNNPMEQVGPGKTETLTITATP  134
```

Identity: 58/118 (49%)
Positives: 67/118 (56%)

Supplementary Figure 9 – Experimentally determined structure of *S. acidocaldarius* AapB vs. Alphafold 2 prediction

a, experimentally-determined structure of AapB (beige) and Alphafold2 prediction of AapB (pink). **b,** superimposition of the experimental and Alphafold-predicted structures. **c,** RMSD between the experimental and Alphafold-predicted structures. **d,** alignment between the sequence predicted by ModelAngelo based on our Aap map, and the sequence for the AapB gene, showing 77% of identity and no gaps along the full length of the 156 amino acids. **e,** alignment between the sequence predicted by Modelangelo and that of the AapA gene, showing only 49% of identity across 134/156 amino acids, omitting the C-terminial residues.

Supplementary Figure 10

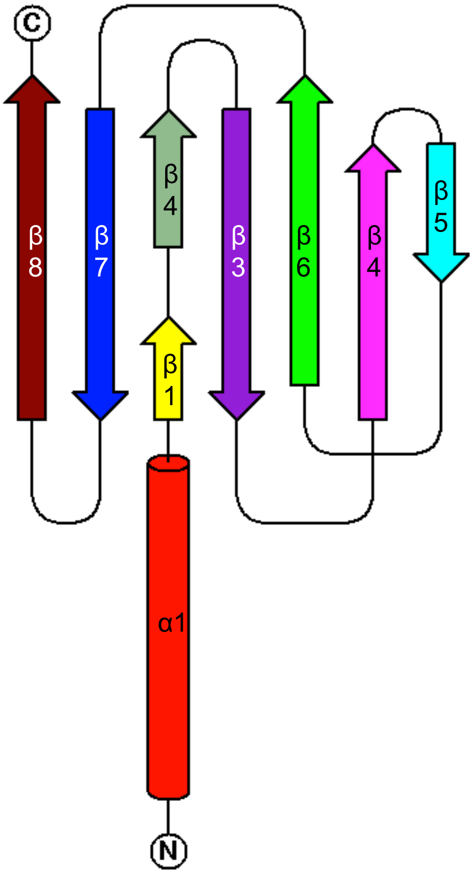

Supplementary Figure 10 – Topology of AapB

Topology diagram of AapB, showing α-helices as cylinders and β strands as arrows.

Supplementary Figure 11

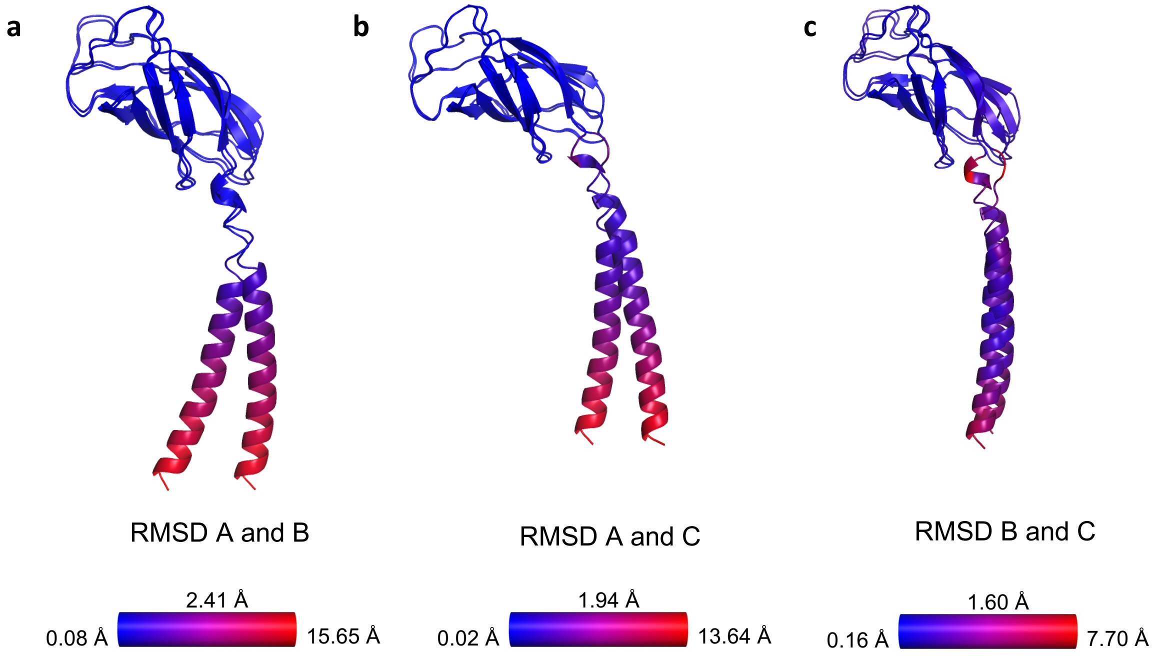

Supplementary Figure 11 – RMSD between the three conformations of AapB
RMSD between conformations A and B **(a)**, A and C (**b**), and B and C **(c)**.

Supplementary Figure 12

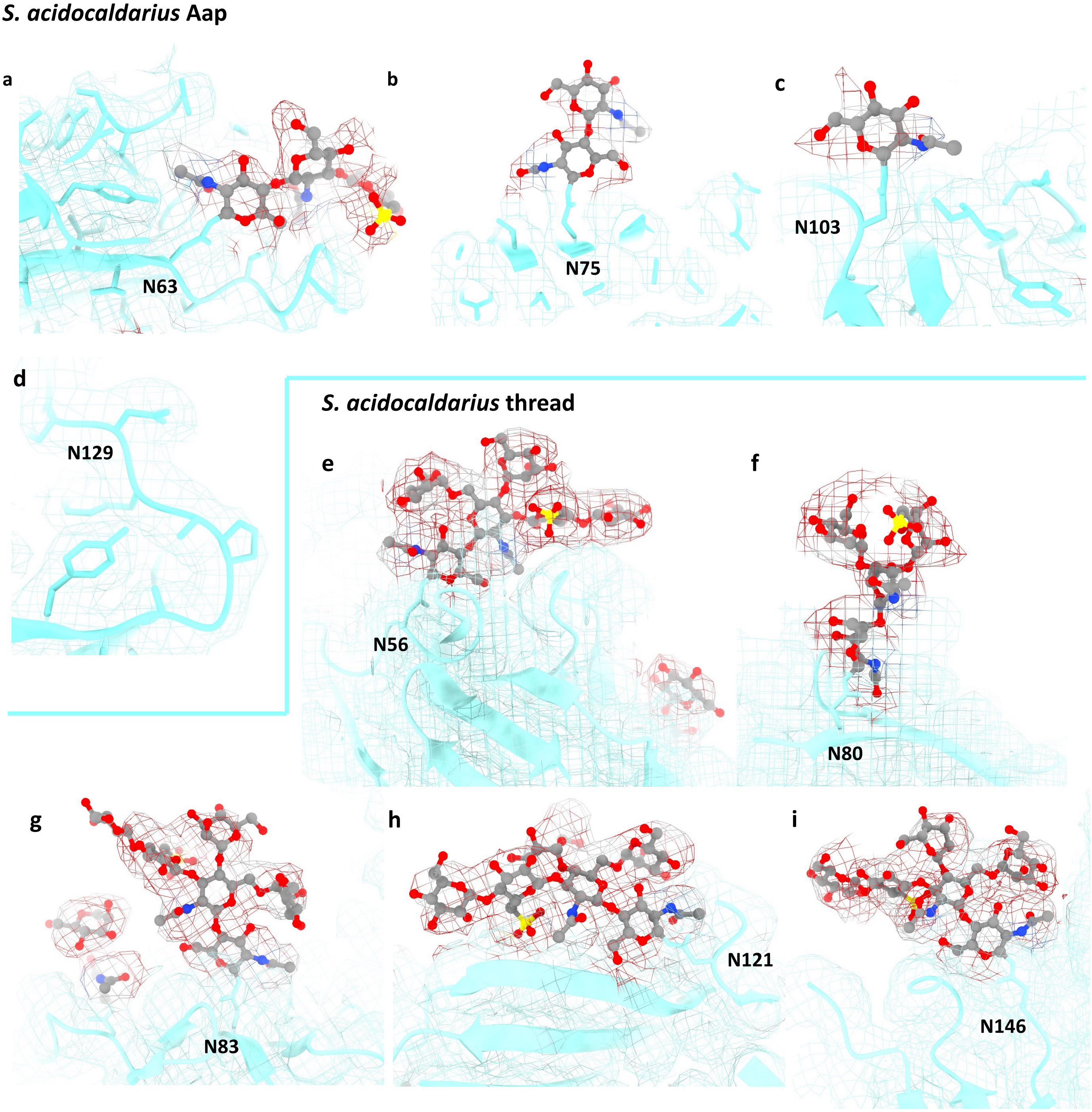

Supplementary Figure 12 – Aap glycosylation

a-c, closeups of the three glycosylation sites (N63, N75 and N103) of AapB. **d,** un-glycosylated asparagine N129. **e-i,** closeups of five glycosylation sites (N56, N80, N83, N121 and N146) of the *S. acidocaldarius* thread filament (PDB: 7PNB) [4]. Protein models are shown as light blue ribbon, map as light blue mesh. Glycans are coloured by element (red, oxygen; blue, nitrogen; grey, carbon; and yellow, sulphur).

Supplementary Figure 13

a

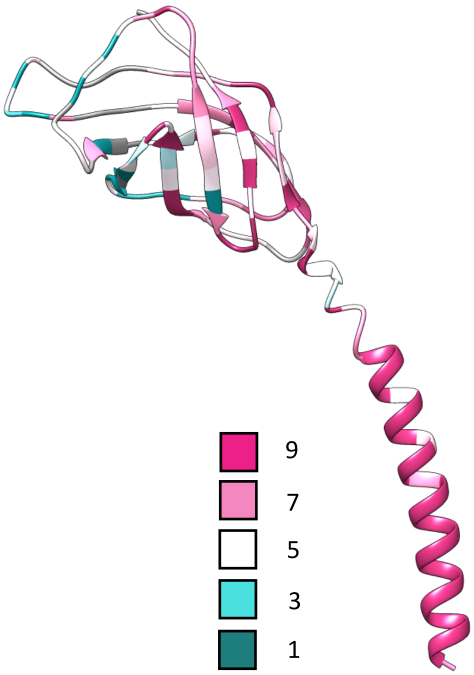

b

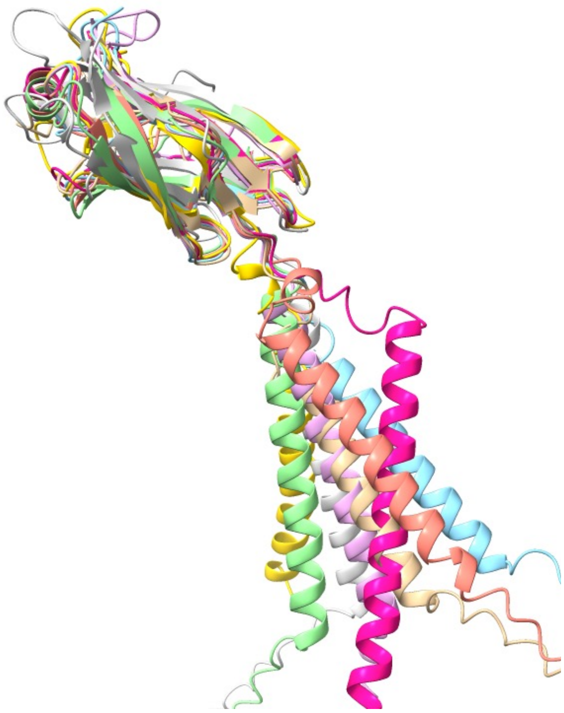

c

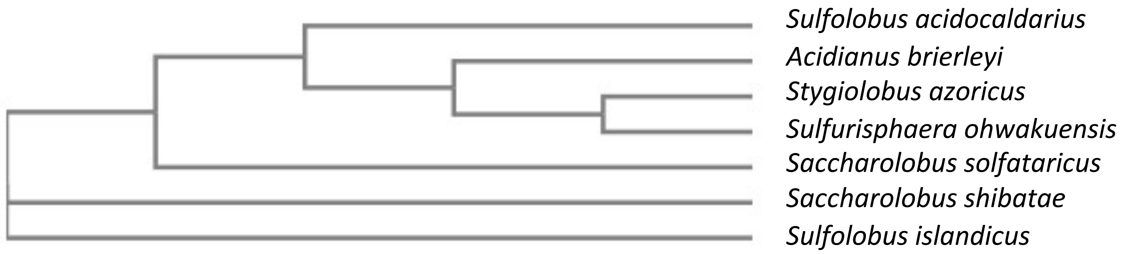

d

```
S.acidocaldarius      MNIEVKKSKKKNMRALSGAIVALILVIAGVIIAIAVVLFAFGLIPGISNQGSIQVLGSGT    60
A.brierleyi           --MKTINRKRRNQKGLSGAVTALILVIASVAIAIVVVGFVFGIFGAVTSTPSVTAAGTGS    58
S.shibatae            ---MKMVSRRKNAKALSGAVTALILVIASVIIALVVVGFAFGLFGAFSGQGTVSQVGTAT    57
S.islandicus          -----MISRRKNAKALSGAVTALILVIASVIIALVVVGFAFGLFGAFTGQGTVTQVGTAT    55
S.solfataricus        ---MDMASRRKNARGLSGAVTALILVIASVIIALVVVGFAFGLFGAFTGQGTVTQVGTAT    57
S.azoricus            --MSLKLKAKRSKKGISGAVTALILVIASVIIALVVVGFAFGLFGAIGGTPTVTQVGTGA    58
S.ohwakuensis         -----MLLRRKAKKGLSGAVTALILVIASVIIALVVVGFAFGLFGAFGGTPTVTQVGTGT    55
                               ::  :.:***:.*******.* **:.** *.**:: .. .  ::   *:.:

S.acidocaldarius      ITNSTASGSSRTIYNITITVKNTGTTSISVTSININGQPFNINGT---APSIPAG-RTQP    116
A.brierleyi           ISEIGN----TGQYTLTVVLDSTGSA--TVVSVSVDGYTTSV------STALSSGNNLIS    106
S.shibatae            LKT-SGT--------LIVTLKNTGANT-QVTGVLINGQSIPLTP----PYTISAGVTTYS    103
S.islandicus          LSASTGT--------LTVTLKNTGATT-QVTGAIINGNPVTLSS----QVTISAGQSTYS    102
S.solfataricus        LSASTLT--------LTVTLKNTGAST-QVTGVLINGNSGSVSG----MTTISAGVNTYT    104
S.azoricus            LYNSTSQ---KGYYEAIITLHSTGNV--QIVSATIVGTSIQVGTSDITGAALSAGANTVT    113
S.ohwakuensis         LTS---S---GNYVVATITLHSTGSV--QIVSATIAGTTYSATS---LSQSLSAGVNTVT    104
                      :                :.:..**     :..  : *             :: :*

S.acidocaldarius      ITFEVTPASGKPNFSPGASYTATIYFSNGQGAPATLIYQG--    156
A.brierleyi           ITLPTG-----FTPTPGQTYTIDLGLNDGQSVAVAAVYTTT-    142
S.shibatae            IALS-GSFTSSLSSLVGSTISLTLQLSNGQTVTVSAIVTS--    142
S.islandicus          ISLGS---SSTLQSLVGSTISLTLQLSNGQTVTVSAIITS--    139
S.solfataricus        ITISIGSISTTLRGLVGSTISLTLILSNGQTVTVSAIVTS--    144
S.azoricus            IYFPTA--AG-FTPQPGSVYQISLGLSDGTTVQVAVTYTGTT    152
S.ohwakuensis         LSFDTS--TGSVTLQPGSTYTISIGLSDGQTVQVAVVYQ---    141
                      : :             *      : :.:*  . .:
```

Supplementary Figure 13 – AapB homologues

a, ConSurf comparison between *S. acidocaldarius* AapB and 6 homologs from related Crenoarchaeota. The model compares the conservation of each predicted structure, where pink indicates maximum and green minimal conservation. On the structural level, the alpha helix and inward-facing β-sheet of the head are the most conserved. **b,** superimposition of all structures shows the structural conservation between all 7 structures. Where no experimental structures were available, AlphaFold2 predictions were used. **c,** phylogenetic relationship between the 7 homologs based on the amino acid sequence. **d,** sequence alignment of the 7 homologs, where the yellow highlighted sequences are predicted N-glycosylation sites.

Supplementary Figure 14

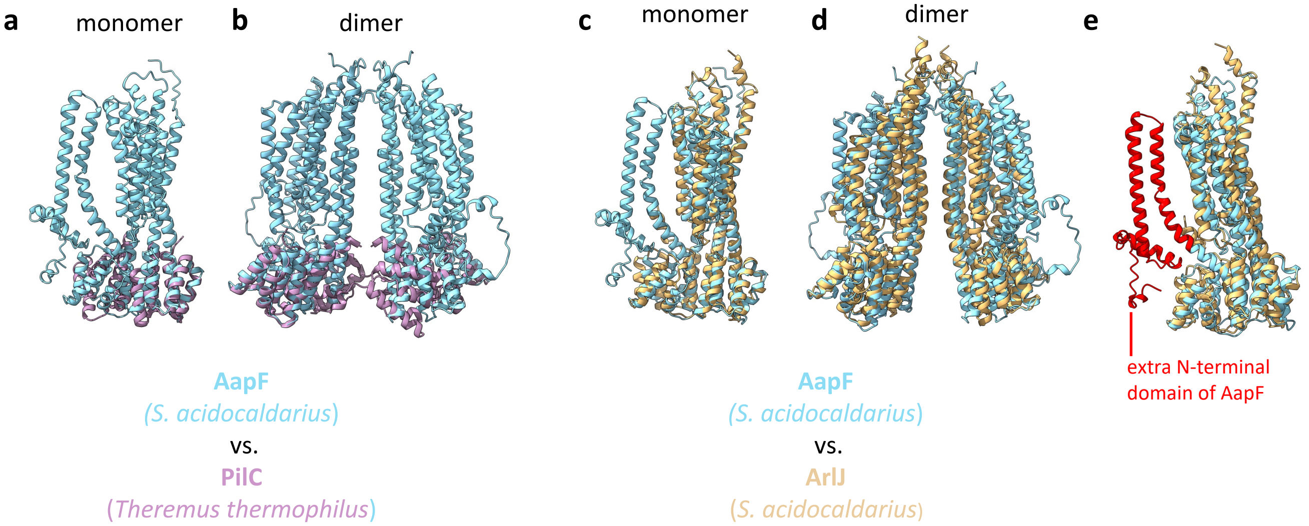

Supplementary Figure 14 – Alphafold prediction of AapF compared to ArlJ, and PilC

a, b, Alphafold2 prediction of an *S. acidocaldarius* AapF (blue) superimposed with the structure of the N-terminal domain of the bacterial PilC from *Thermus thermophilus* (pink; PDB-2WHN,[5]) as a monomer (a) and dimer (b). **c, d,** Alphafold2 prediction of *S. acidocaldarius* AapF (blue) superimposed with the AlphaFold2 model of *S. acidocaldarius* ArlJ (beige) as a monomer (c) and a dimer (d). **e,** comparison between the Alphafold models of *S. acidocaldarius* AapF (blue) and ArlJ (beige). The extra N-terminal domain of AapF is highlighted in red.

Supplementary Figure 15

a

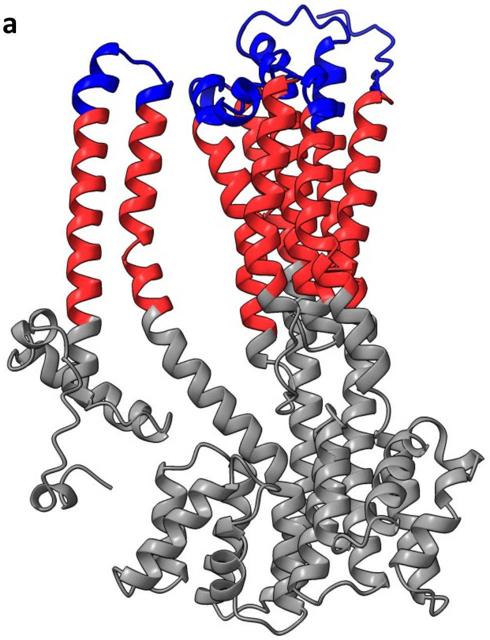

b

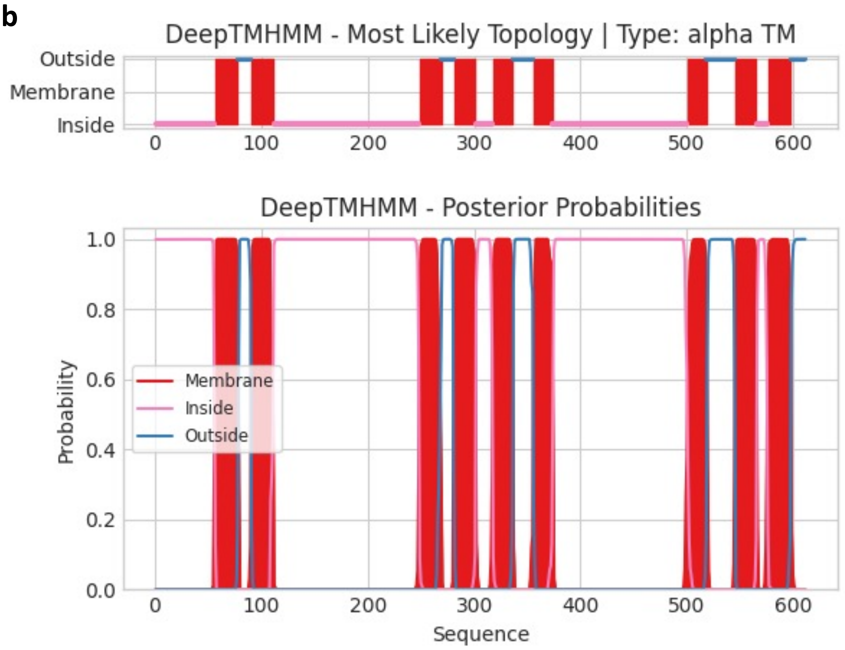

Supplementary Figure 15 – Topology prediction for *S. acidocaldarius* AapF

a, Alphafold model of the AapF monomer coloured by topology, as predicted by DeepTHMHH. Blue, cell-external; red, membrane-integral; grey cytosolic. **b,** DeepTMHMM probability plots for the predicted topology.

Supplementary Figure 16

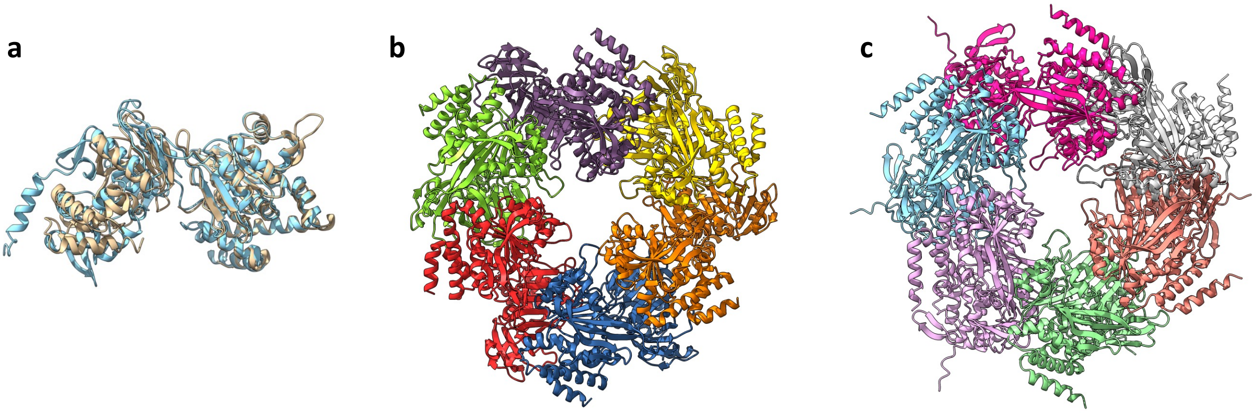

Supplementary Figure 16 – Alphafold prediction of AapE compared with ArlI

a, superimposition of the AlphaFold2 predicted structure of *S. acidocaldarius* AapE and the X-ray structure of *S. acidocaldarius* Arl (PDB-4II7,[6]). **b, c,** hexameric models of ArlI (b) and AapE (c).

Supplementary Figure 17

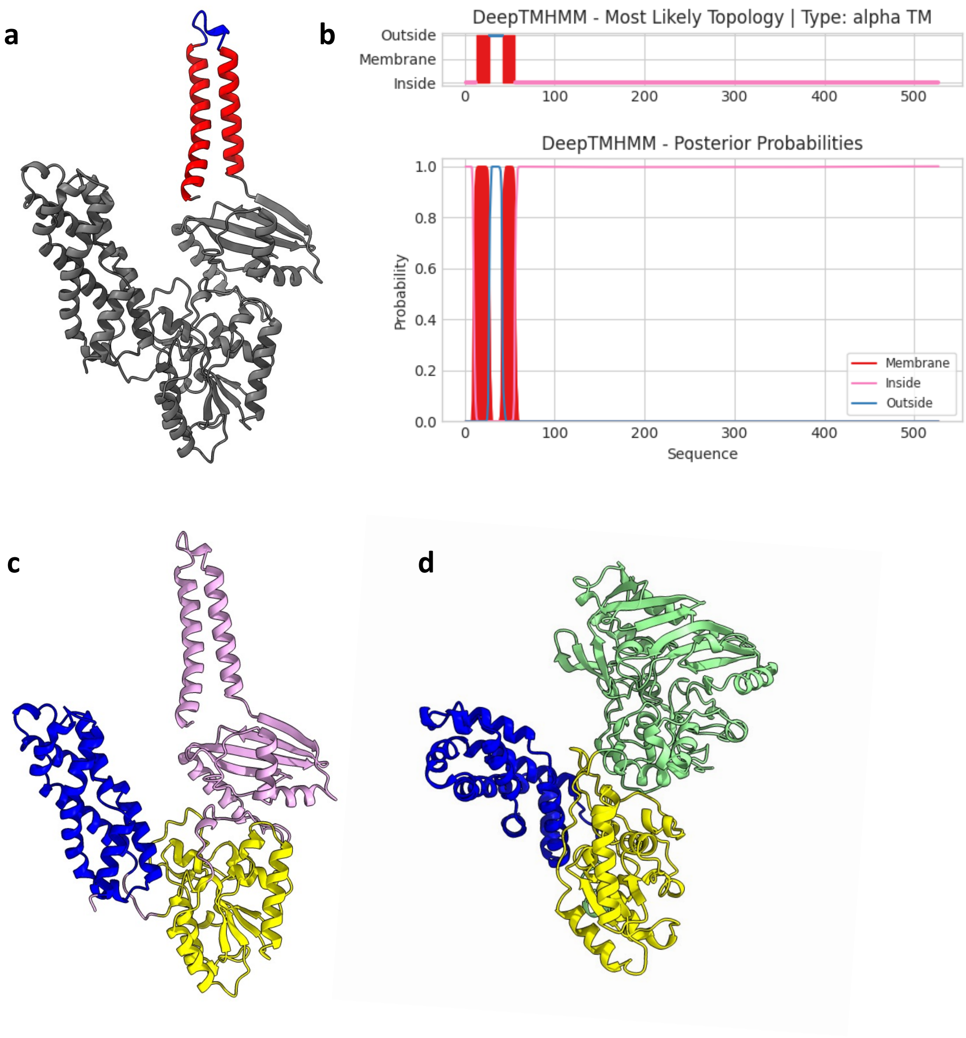

Supplementary Figure 17 – Alphafold prediction of AapX

a, Alphafold model of the AapX monomer coloured by topology, as predicted by DeepTHMHH. Blue, red and grey represent cell-external, membrane-integral and cytosolic, respectively. **b,** DeepTHMHH probability plots for the predicted topology in (a)**. c**, the three distinct domains of *S. acidocaldarius* AapX in blue, yellow and pink. "Comparing *S. acidocaldarius* AapX (c) with homologues, e.g. the FAD binding protein of *Saccharolobus solfataricus*[3] (d) shows that the FAD domain (green in d) is exchanged for a membrane-binding domain in *S. acidocaldarius* (pink in c)."

Supplementary references

1. Wurtzel, O. *et al.* A single-base resolution map of an archaeal transcriptome. *Genome Res* **20**, 133–141 (2010).
2. Meyer, B. H. et al. Agl16, a thermophilic glycosyltransferase mediating the last step of N-Glycan biosynthesis in the thermoacidophilic crenarchaeon sulfolobus acidocaldarius. J Bacteriol 195, 2177–2186 (2013).
3. Henche, A. L. et al. Structure and function of the adhesive type IV pilus of Sulfolobus acidocaldarius. Environ Microbiol 14, 3188–3202 (2012).
4. Gaines, M. C. et al. Electron cryo-microscopy reveals the structure of the archaeal thread filament. Nature Communications 2022 13:1 13, 1–13 (2022).
5. Karuppiah, V., Hassan, D., Saleem, M. & Derrick, J. P. Structure and oligomerization of the PilC type IV pilus biogenesis protein from Thermus thermophilus. Proteins: Structure, Function, and Bioinformatics 78, 2049–2057 (2010).
6. Reindl, S. et al. Insights into FlaI Functions in Archaeal Motor Assembly and Motility from Structures, Conformations, and Genetics. Mol Cell 49, 1069–1082 (2013).
