# Supplementary material for: CryoEM reveals the structure of an archaeal pilus involved in twitching motility
Source: Nat Commun. 2024 Jun 14;15:5050. doi: 10.1038/s41467-024-45831-w (PMC11178815; doi:10.1038/s41467-024-45831-w)
Supplement: Supplementary file 3 — Description of Additional Supplementary Files [file 41467_2024_45831_MOESM3_ESM.pdf]

Description of Additional Supplementary Files:

Supplementary Movie 1: Morph between the three AapB conformations (A, B and C)

Supplementary Movie 2: Flexibility analysis of the Aap and thread filament.
